# A surface treatment method for improving the attachment of PDMS: acoustofluidics as a case study

**DOI:** 10.1038/s41598-023-45429-0

**Published:** 2023-10-24

**Authors:** Abdulla Al-Ali, Waqas Waheed, Fadi Dawaymeh, Nahla Alamoodi, Anas Alazzam

**Affiliations:** 1 Mechanical Engineering Department, Khalifa University, Abu Dhabi, United Arab Emirates; 2 System on Chip Lab, Khalifa University, Abu Dhabi, United Arab Emirates; 3 Chemical Engineering Department, Khalifa University, Abu Dhabi, United Arab Emirates

**Keywords:** Chemistry, Biomedical engineering, Chemical engineering, Mechanical engineering

## Abstract

A method for a permanent surface modification of polydimethylsiloxane (PDMS) is presented. A case study on the attachment of PDMS and the lithium niobate (LiNbO_3_) wafer for acoustofluidics applications is presented as well. The method includes a protocol for chemically treating the surface of PDMS to strengthen its bond with the LiNbO_3_ surface. The PDMS surface is modified using the 3-(trimethoxysilyl) propyl methacrylate (TMSPMA) silane reagent. The effect of silane treatment on the hydrophilicity, morphology, adhesion strength to LiNbO_3_, and surface energy of PDMS is investigated. The results demonstrated that the silane treatment permanently increases the hydrophilicity of PDMS and significantly alters its morphology. The bonding strength between PDMS and LiNbO_3_increased with the duration of the silane treatment, reaching a maximum of approximately 500 kPa. To illustrate the effectiveness of this method, an acoustofluidic device was tested, and the device demonstrated very promising enhanced bonding and sealing capabilities with particle manipulation at a flow rate of up to 1 L/h by means of traveling surface acoustic waves (TSAW). The device was reused multiple times with no fluid leakage or detachment issues. The utility of the presented PDMS surface modification method is not limited to acoustofluidics applications; it has the potential to be further investigated for applications in various scientific fields in the future.

## Introduction

Polydimethylsiloxane (PDMS) is a popular elastomeric material in science and engineering, particularly in biomedical engineering due to its availability, transparency, biocompatibility, and simple fabrication^1–3^. Despite PDMS’s compatibility with different engineering applications, its hydrophobic nature acts as a barrier to multiple applications. It resists fluid flow and weakens the adhesion between PDMS and other surfaces. In fact, it is hard to bond PDMS to other materials without proper surface treatment and sealing techniques^2, 3^.

In the field of microfluidics, a prominent branch of bioengineering that has gained substantial traction since its inception in the 1970s, several advantages, such as low sample volume requirements, environmental friendliness, and laminar flow within microchannels have propelled its growth^4–6^. As a branch of microfluidics, the acoustofluidics field primarily relies on piezoelectricity as the source of mechanical waves^7–9^. A commonly employed piezoelectric material for acoustofluidic applications is LiNbO_3_ ceramic, which comes in various types based on rotations and cuts^10^. Among these, the Y-rotated X-propagating 128°-LiNbO_3_ is the best suited for surface acoustic waves (SAW) generation and propagation due to its high electromechanical coupling coefficient and high electric potential strength^9^. Another advantage is that LiNbO_3_ is a transparent wafer, facilitating easy microscopy processes and monitoring^11^. Its Curie temperature is 1210 °C, meaning it will not lose its polarization unless exposed to a very high temperature^9^. However, its temperature stability is very low^12^. Furthermore, LiNbO_3_ exhibits the most efficient SAW propagation along the x-axis, with other directions resulting in less efficient propagation and potentially generating undesired wave types^13,14^. The generated SAW has a velocity on the surface of the LiNbO_3_ wafer of about 3990 m/s^11^. In practice, the combination of a LiNbO3 wafer and a PDMS microchannel is common in SAW-actuated microfluidic platforms. This setup typically involves bonding a PDMS microchannel to a LiNbO_3_ wafer containing interdigital transducers (IDT) for SAW production^15,16^. It is essential to emphasize that the bonding process plays a pivotal role in achieving proper sealing and stable operations, and this is where the hydrophobic nature of PDMS presents a notable challenge^2^.

In microfluidics, various bonding mechanisms are available, including anodic, fusion, thermal, adhesive, plasma, ultrasonic welding, solvent, and surface bonding. However, due to factors such as high-temperature requirements, bonding strength, biocompatibility, or the temporary nature of the resulting bond, the majority of these methods are not preferred within the acoustofluidics domain. Typically, two primary bonding methods dominate in microfluidics: adhesive bonding and surface bonding. Adhesive bonding involves using an adhesive layer between two surfaces to ensure a secure bond. However, it is sensitive to micro-level precision and biocompatibility concerns, limiting its application in this field. In contrast, surface bonding is extensively employed, with researchers employing various protocols, surface treatments, and procedures, each with its own advantages and disadvantages^3, 17^.

Researchers used adhesive bonding and surface bonding methods to bond PDMS and LiNbO_3_ wafer in acoustofluidic devices. However, a layer that enhances the bonding properties of the material has been used in place of the adhesive layer. Ma et al.^18^ have used a very thin PDMS layer to enhance the adhesion between the PDMS part and the LiNbO_3_ wafer in their TSAW-based particle separation device. Mutafopulos et al.^19^ introduced a layer of SiO_2_ to facilitate the bonding of the PDMS on the piezoelectric wafer. Ma et al.^20^ added a similar SiO_2_ layer to encourage adhesion and protect the IDT beneath. However, these extra layers affect the attenuation factor negatively. For most applications, a reduction in the acoustic force is unfavorable. For acoustofluidics, Oxygen^21^ and air plasma^22^ were both used to temporarily bond the LiNbO_3_ wafer with the PDMS part. Most recently, Guo et al.^23^ introduced the oxygen–nitrogen dual plasma surface modification method. The resulting device can withstand higher flow rates and has a higher bonding strength compared to the oxygen plasma method. However, plasma enables only a temporary enhancement on the treated surface for a few minutes^2^. Higher flow rate applications necessitate improvements in bonding and sealing technology to withstand high pressure and enable a permanently enhanced surface.

Several studies have investigated the potential of chemical surface treatment to improve the bonding of PDMS to other surfaces. Sunkara et al.^24^ used oxygen plasma followed by a 1% (3-Aminopropyl) triethoxysilane (APTES) surface modification reagent to bond PDMS with different thermoplastics. This method requires only a few minutes of processing, and it showed a modification in bonding strength. The reported bonding strength was in the range of 400 kPa. The same group used a similar methodology to bond PDMS with metals and plastics using oxygen plasma, followed by a 1% APTES treatment. The method successfully increased the bonding ability of the targeted metals and plastics, with varying bonding strengths of each material^25^. Gu et al.^26^ used corona discharge and TMSPMA silane to bond PDMS with thermoplastics. However, this method requires overnight processing to achieve an acceptable bond. Zhu et al.^27^ used oxygen plasma followed by 5% APTES treatment to enhance bonding between PDMS and SU-8. The proposed method requires only 30 min of treatment, and the resulting bonding strength can go up to 1400 kPa.

In this paper, a novel surface treatment method using TMSPMA and a detailed implementation protocol are presented to enhance the bonding between the LiNbO_3_ wafer and the PDMS microchannel. TMSPMA silane was used at room temperature to modify the surface of the PDMS before being bonded to the LiNbO_3_ wafer to build hermetic acoustofluidics. Permanent changes to the surface of the PDMS microchannel were introduced to promote the sealing of the microchannel even at comparably high flow rates. The method does not require any adhesive layers to be present, and the entire surface treatment process can be conducted within 60 min. Several tests have been conducted to compare the surface hydrophobic nature, surface roughness, bonding strength, and surface chemical composition between the bare PDMS samples and the treated ones. The tests were followed by the successful manipulation of polystyrene (PS) particles via TSAW and a microchannel leakage test. According to the results, the treated sample showed enhanced bonding between the PDMS and the LiNbO_3_. The permanent surface treatment enabled multiple uses of the microchannel without any leakage. The newly introduced method intends to solve the bonding issue between the LiNbO_3_ wafer and PDMS microchannel to pave the way for further development in the acoustic microfluidics field. Consequently, the method can prove influential in developing high-throughput acoustofluidics-based devices and thus enabling them to be applied in actual clinical settings.

## Materials and protocol

### Silane bonding agents

Typical surface treatment methods to improve the attachment of PDMS and other substrates include surface activation using oxygen plasma, corona plasma, and UV/ozone treatment, the use of adhesives, and the use of chemical bonding agents. Chemical bonding agents, or chemical adhesion promoters, are chemicals that promote the adhesion of two materials at their interfaces, for example, at the interface between an organic polymer and an inorganic surface. In general, silane adhesion promoters consist of four substituents bonded to a single silicon atom. There are three reactive alkoxy groups, methoxy or ethoxy, and one organic group. Silane coupling agents serve as adhesion promoters or bridging agents between inorganic and organic substrates in the interphase region, which increases the adhesion between the two dissimilar materials^28^. For this purpose, TMSPMA silane was used, which was applied to the PDMS substrate to improve the adhesion between the PDMS and LiNbO_3_ wafer.

### Micro fabrication and surface treatment protocol

**Figure 1:**
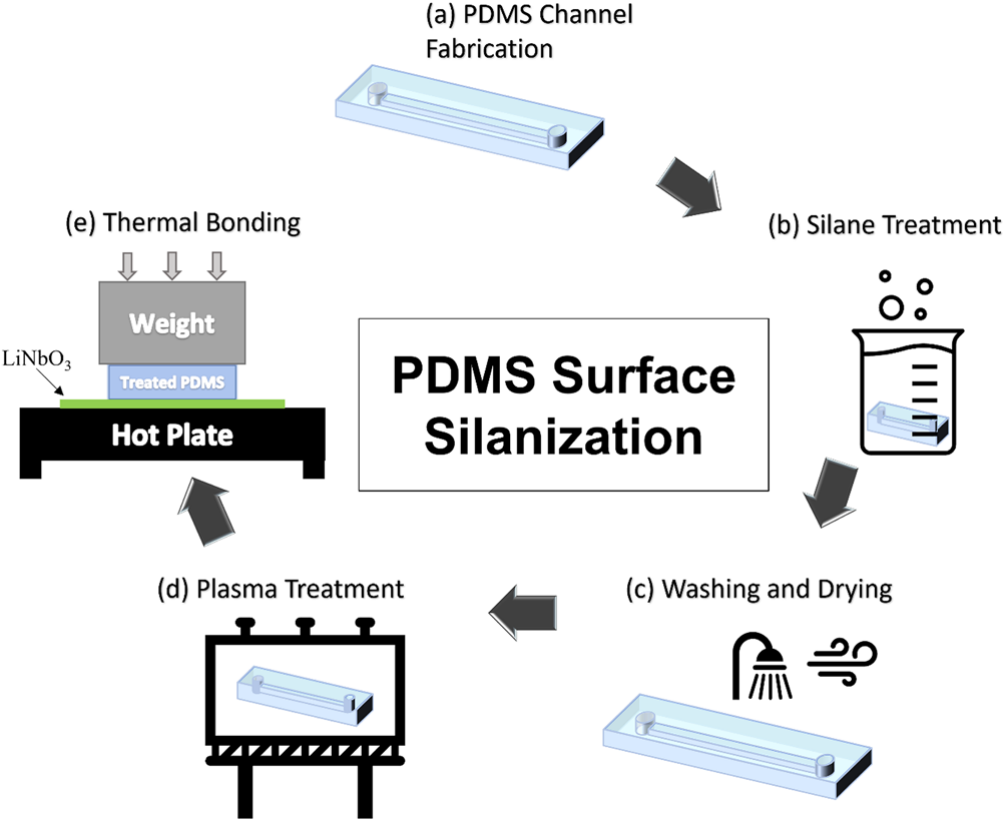
The implementation protocol of PDMS surface treatment using TMSPA to change the surface properties of the PDMS through five steps, which are (**a**) PDMS channel fabrication, (**b**) silane treatment, (**c**) washing and drying, (**d**) plasma treatment, and (**e**) thermal bonding.

The primary purpose of the surface silanization is to permanently change the surface properties of the PDMS. This change is required to ensure better adhesion between the PDMS and the LiNbO_3_ wafer and prevent leakage once the device is in operation mode. The treatment should also enable the microdevice to be used multiple times. In this work, the attachment of PDMS to LiNbO_3_ involves five steps: PDMS channel fabrication, TMSPMA silane treatment, washing and drying, plasma treatment, and thermal bonding, as illustrated in Fig. 1. To examine the effect of silanization on PDMS, multiple samples were treated with silane for varying time periods and then tested. Each sample has been labeled according to Table 1. The labels used to represent each sample in Table 1 will be used throughout the work.

**Table 1:** Samples labels used to represent each sample throughout the entire work for accuracy and consistency.

#	Sample label	Silanization time (min)
1	S-0	0
2	S-30	30
3	S-60	60
4	S-120	120
5	S-180	180
6	S-240	240

#### PDMS channel fabrication

A PDMS (SYLGARD 184 Silicone Elastomer from DOW chemical company) part with a microchannel is fabricated using standard soft lithography process. A master mold is created on a silicon wafer using SU-8 (negative photoresists from MICRO-CHEM) and then used as a PDMS replica mold. Many published works have already described the steps involved in making a microchannel using PDMS^29, 30^.

#### Silane treatment

To prepare the silane solution, a mixture of ethanol (88 wt.%) (from Fisher Scientific UK), deionized water (6 wt.%), and TMSPMA (6 wt.%) (TMSPMA 98% from Sigma-Aldrich) is prepared based on literature^3^. The solution is prepared by mixing ethanol and deionized water, and then the TMSPMA is added. A beaker is used to immerse the previously prepared PDMS microchannel in the silane solution. PDMS must be completely submerged in the silane solution, and the glass beaker with the sample and silane must be carefully covered and sealed to prevent any evaporation. Six representative samples prepared using the same method but salinized for different time durations were used to assess the silanization effect, as shown in Table 1.

#### Washing and drying

After the silanization step, ethanol is used to wash the samples. This step ensures that any remaining silane is removed from the PDMS part. As the presence of silane in the microchannel during experiments may affect the results and raise safety concerns, the washing procedure is repeated multiple times to ensure the complete removal of excess silane. Following this washing step, compressed nitrogen is used to dry the treated PDMS microchannel. If the microchannel inlets and outlets were not pierced during fabrication, the holes can also be made after silanization.

#### Plasma treatment

PDMS is hydrophobic by nature, so it must be treated with oxygen/argon plasma before it can be bonded to a LiNbO_3_ wafer^31^. Although TMSPMA silanization improved the hydrophilicity of the PDMS surface in a permanent manner, oxygen plasma treatment has been proven in this experiment that it can further improve the hydrophilicity of the PDMS surface temporarily.

#### Thermal bonding

The PDMS microchannel is thermally bonded to the LiNbO_3_ wafer as the final step in the attachment process. Following the plasma treatment of both the PDMS microchannel and the LiNbO_3_ wafer, a drop of ethanol is placed on the wafer to facilitate the alignment of the PDMS microchannel at the desired location on the LiNbO_3_ wafer. The alignment is performed using a microscope, after which the device is left at room temperature for a few minutes to allow the ethanol to evaporate. The PDMS microchannel and LiNbO_3_ wafer are then heated at 90 °C for 4 hours with a weight element of 1–2 kg placed on top of them. This process strengthens the bond between the two parts. The heating process is then terminated, and the device is gradually cooled to room temperature prior to use.

## Results and discussion

Several assessments were carried out to study the effect of silanization on the surface of PDMS as well as the attachment of PDMS to LiNbO_3_. Results from these assessments are presented in the following sections.

### Microscopic imaging

**Figure 2:**
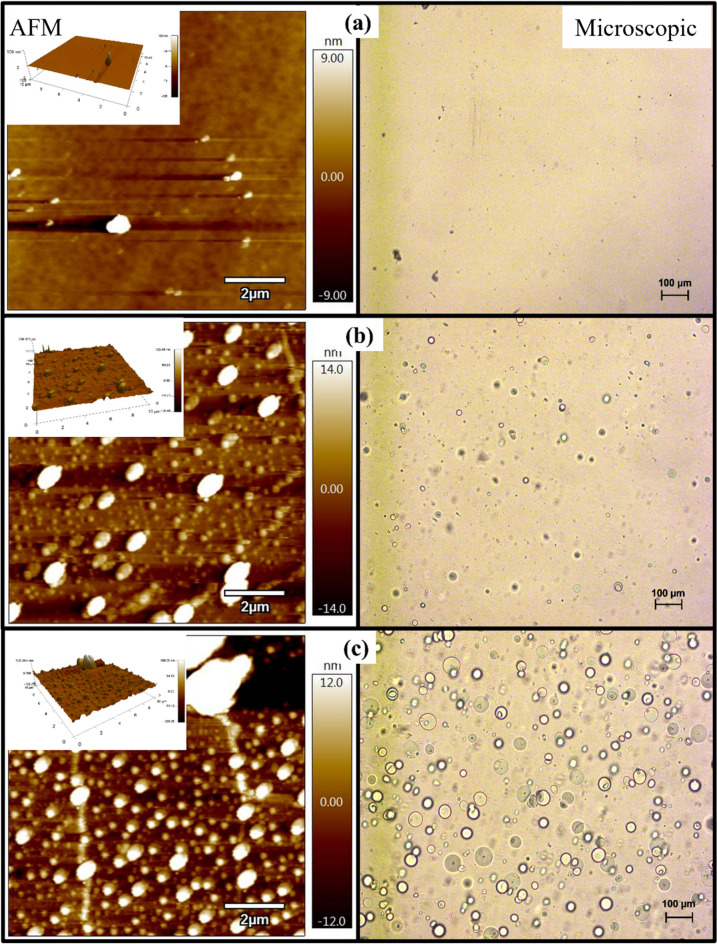
An AFM (lift) and microscopic (right) examination of three representative samples (**a**) S-0, (**b**) S-60, (**c**) S-180.

As depicted in Fig. 2 (a–c, right-side), three of the six representative samples examined under a microscope are shown. The microscopic images clearly show the effect of silanization on the surface morphology of PDMS (extra images are available in the supplementary material). Figure 2a depicts the surface of an untreated sample (S-0). The S-0 surface is devoid of any visible surface characteristics. Tiny, mound-shaped defects are depicted for the S-30 sample, which was treated for only 30 min. It indicates that the silanization process has just begun to affect the surface morphology of the PDMS. Figure 2b depicts microscopic images of the surface of an S-60 sample. The formation of clear mounds indicates that the silanization process impacts the PDMS surface and changes its morphology. For S-120, which underwent a two-hour silanization process, The mounds are significantly larger than those observed on the surface of S-60. In Fig. 2c, it is evident that the density and size of the mounds increase as the silanization period increases. S-180 PDMS samples have a significantly higher mound density than S-120 samples, and the same is true for S-240 compared to S-180. It is clear from the microscopic images that the silanization process has successfully altered the surface morphology of the PDMS. However, additional testing is needed to get a clear picture of the PDMS surface morphology following the silanization process.

### Atomic force microscopy 

**Figure 3:**
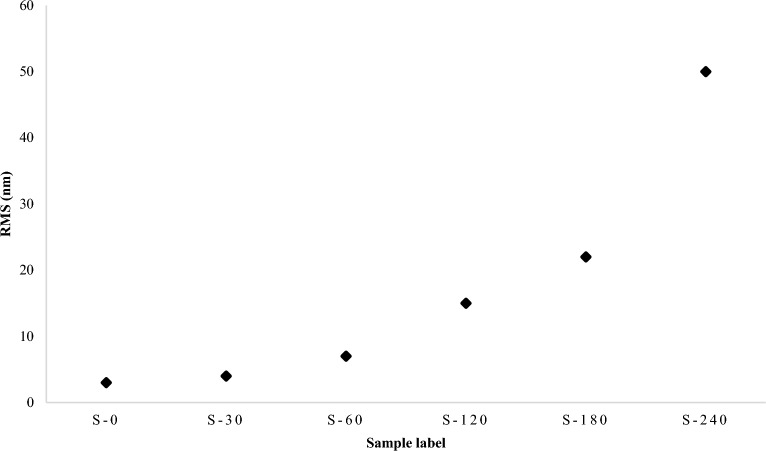
The RMS values chart that represents the surface height variation for the 6-representative samples.

The atomic force microscopy (AFM) is the second examination that is performed. The AFM is used to examine the PDMS's surface topography after the silanization process. AFM provides valuable information and an indication of changes in the surface topography of the PDMS sample, which directly affect its wettability and bond strength to other materials. Figure 2 illustrates the AFM microscopy results for three samples of the six cases. The results of AFM microscopy are consistent with those of microscopic imaging in terms of the general effect of the silane on the PDMS surface. In Fig. 2a, the AFM results for the untreated PDMS sample, S-0, do not reveal any surface variation or roughness. A minor change in the surface morphology was observed in S-30. This indicates that the silanization process begins to affect the surface roughness of the PDMS after 30 min. Figures 2b and c illustrate the changes in surface morphology, and the results demonstrate the effect of silanization time on the PDMS surface. The root mean square (RMS) value for the PDMS surface morphology is shown in Fig. 3. The RMS, which represents the number of peaks and valleys, or surface height variation created in the PDMS surface after the silanization process, provides a clear indication of the extent to which the silanization process can affect the PDMS surface morphology. As shown in Fig. 3, the RMS value increases significantly after three hours of silanization. It is important to note that the surface of the S-240 sample was clearly curved, as determined by a visual assessment of the sample. The swelling issue has been reported previously in multiple studies^32^.

### Wettability test

**Figure 4:**
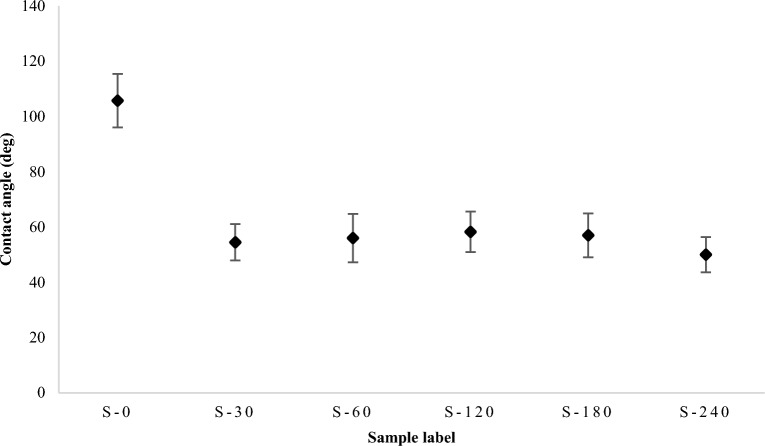
Chart of the average contact angles of the wettability test conducted for the 6-representative samples.

**Figure 5:**
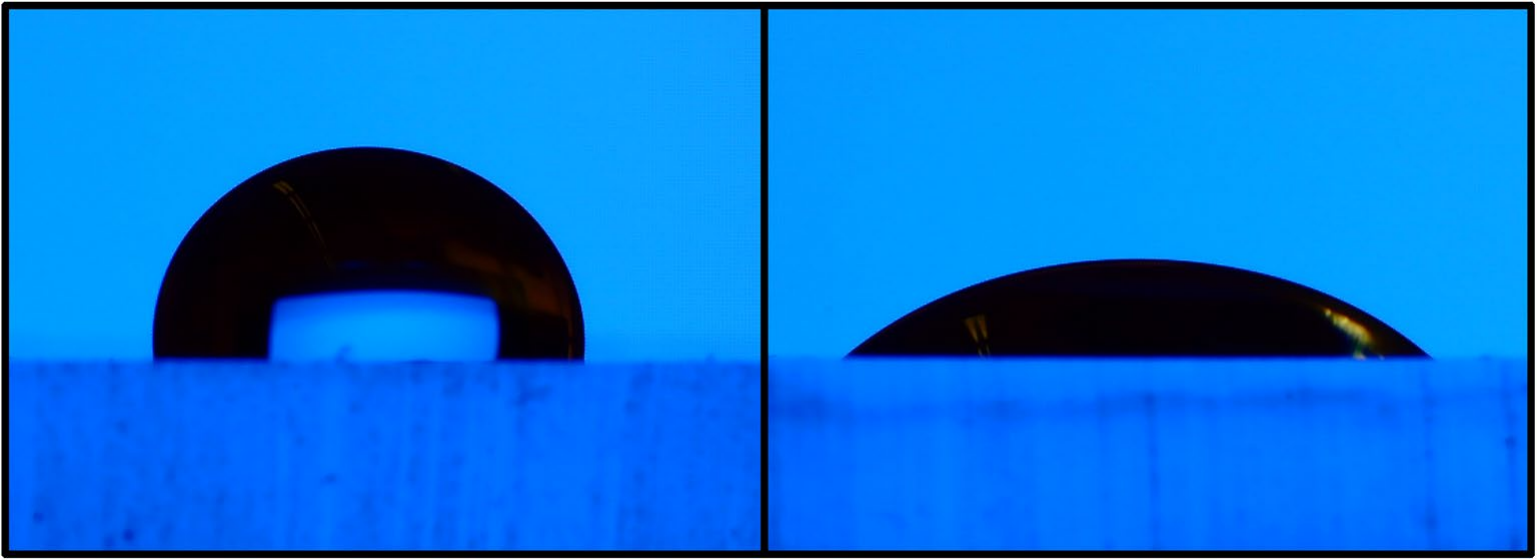
An illustration of the difference in the contact angle of the treated sample (S-60) on the right side compared to the bare PDMS sample (S-0) on the left side.

The surface energy of a microfluidic device is crucial to a number of operations. In this section, the effect of the silanization process on the surface wettability is investigated. Hydrophilic PDMS microchannels have several advantages in biological applications and should enhance the adhesion of PDMS to various surfaces. To determine the effectiveness of the silanization process on wettability, the contact angle of six representative samples was measured with a goniometer using the sessile drop method. A high-resolution camera-equipped optical subsystem was used to capture the profile of the sessile drop and then calculate the contact angle. The wettability test results are shown in Fig. 4. After 30 min of silanization, the contact angle has decreased dramatically, as shown in the figure. This demonstrates that the salinization process improves the wettability of the PDMS surface. It is also important to note that the duration of silanization has no significant effect on the contact angle. Contact angles from wettability tests showed high consistency between the five treated samples (excluding S-0). Figure 5 depicts the contact angle for the treated sample (S-60) and the untreated PDMS sample (S-0). The figure shows that the salinization process modifies the wettability of PDMS and improves its ability to support different applications in the microfluidic field. It’s important to note that the wettability test was performed directly after the silanization process and again after 3 days, revealing similar results. This indicated that the change in wettability caused by this silanization permanently impacts the PDMS surface’s wettability.

### Fourier-transform infrared spectroscopy

**Figure 6:**
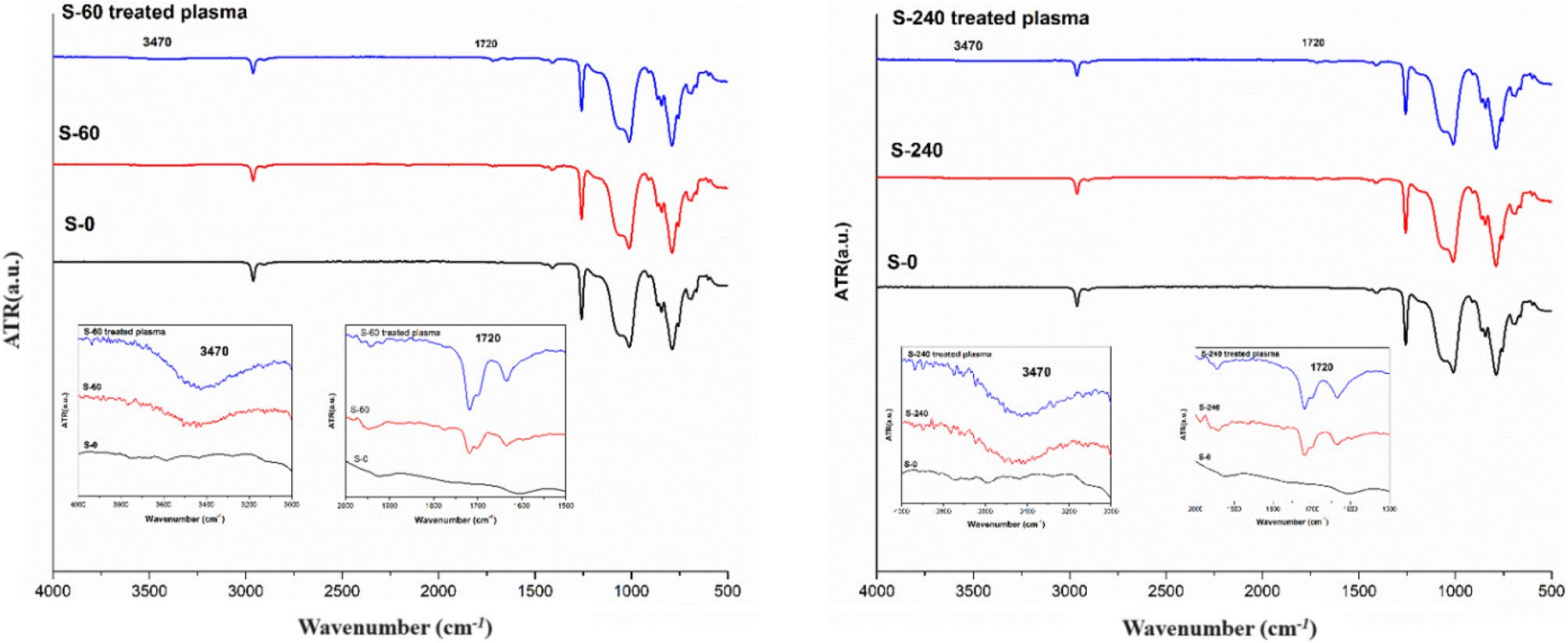
FTIR-AR spectra of (left side) S-0, S-60 and S-60 treated with plasma, (right side) S-0, S-240 and S-240 treated with plasma.

**Figure 7:**
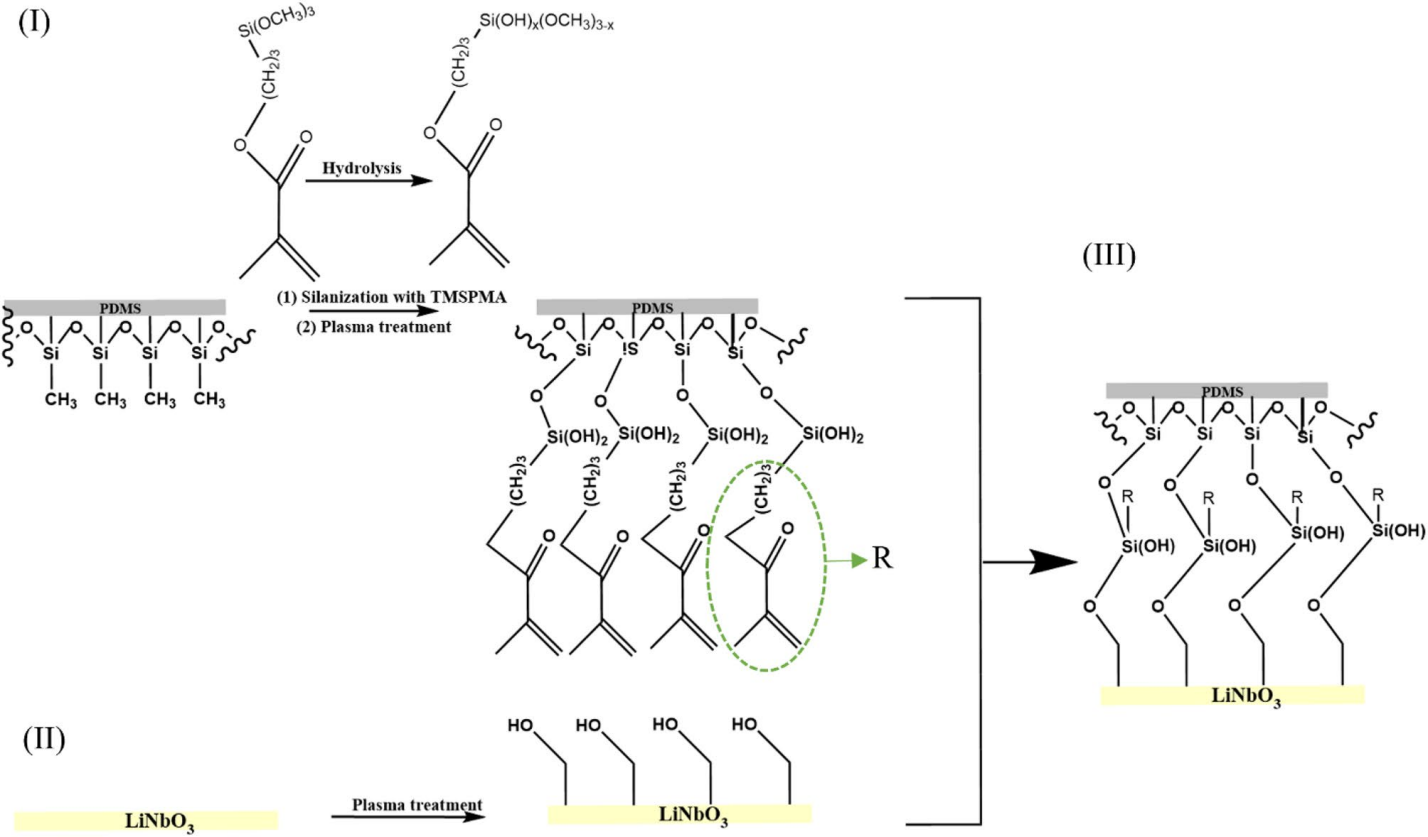
Schematic representation of the proposed mechanism for the bonding of PDMS and LiNbO_3_. (I) Attachment of TMSPMA to PDMS through the bonding between OH groups of hydrolyzed TMSPMA and Si–CH3 groups in PDMS forming –Si–O–Si– bond. Formation of C–O and C=O due to the breaking of C=C bonds in TMSPMA after plasma treatment, while unhydrolyzed Si–OCH3 were transformed to Si–OH. (II) Generation of OH groups on LiNbO_3_ surface by activation with plasma. (III) Bonding of PDMS and LiNbO_3_ by the formation of a covalent bond via the condensation reaction between Si–OH groups on the PDMS surface and OH groups on the LiNbO_3_ surface.

The Fourier-transform infrared (FTIR) spectra of S-0, S-60, S-60 treated with plasma, S-240, and S-240 treated with plasma are shown in Fig. 6. The samples were studied to compare the chemical groups on the PDMS surface and to analyze the changes of the functional groups on the PDMS surface during silanization with TMSPMA and plasma treatments. The FTIR spectra of the S-0 substrate (Fig. 6) show a peak at 790 cm^−1^ for the CH3 rocking and Si–C stretching modes in Si-CH3, peaks in the range of 950–1150 cm^−1^ for the stretching mode of Si–O–Si, a peak at 1258 cm^−1^ for the symmetric CH3 bending in Si-CH3, and a peak at 2962 cm^−1^ for the C–H stretching in CH3, in agreement with the literature^33–35^. The peaks at 1720 and 1640 cm^−1^ for S-60 and S-240 correspond to the deformation of C=O and C=C groups present in the structure of TMSPMA, respectively, indicating a successful modification process. The absorption peak near 3470 cm^−1^ corresponds to the presence of –OH groups formed during the hydrolysis of methoxy (OCH3) groups during the silanization process. Oxygen-containing groups such as carbonyl and hydroxyl groups formed during the silanization process are the main reason for the reduction of the contact angle and the improvement of the hydrophilicity of PDMS surfaces. Compared to S-60 and S-240, the intensity of oxygen-containing groups increases with plasma treatment. This can be attributed to the ability of plasma to break C=C bonds in TMSPMA and form new C–O and C=O groups^36^. In addition, the plasma can also transfer unhydrolyzed Si-OCH3 to Si-OH in this step of surface modification^4^. This increase in the concentration of oxygen-containing groups after plasma treatment could be the reason for the improvement in the adhesion between the PDMS microchannel and the LiNbO_3_ wafers. Figure 7 illustrates the proposed mechanism of bonding PDMS and LiNbO_3_ substrates.

### Bonding strength measurement (tensile test)

**Figure 8:**
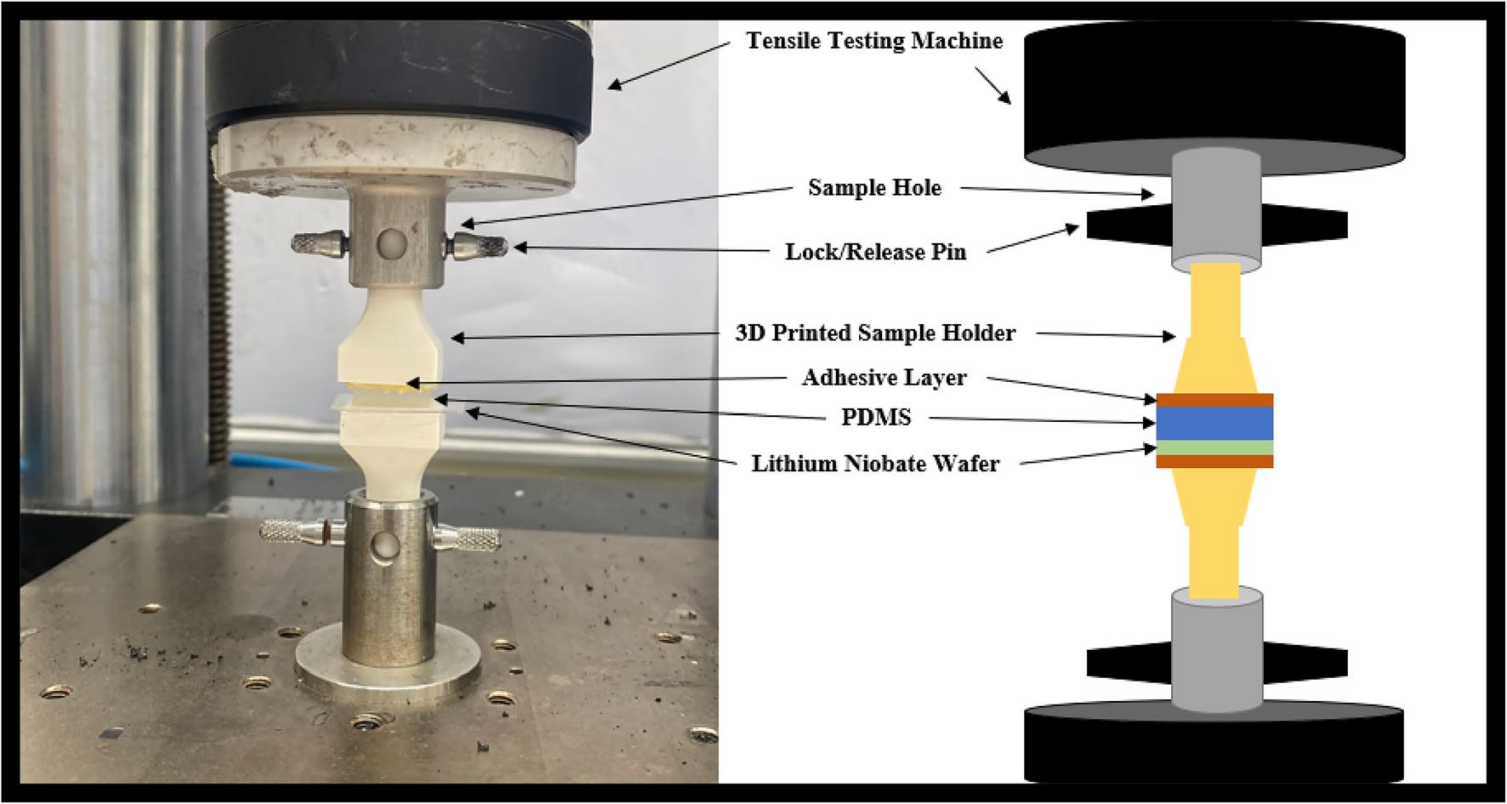
Right side: schematic illustration of the tensile test setup. Left side: tensile testing machine loaded with one of our samples for tensile testing.

**Figure 9:**
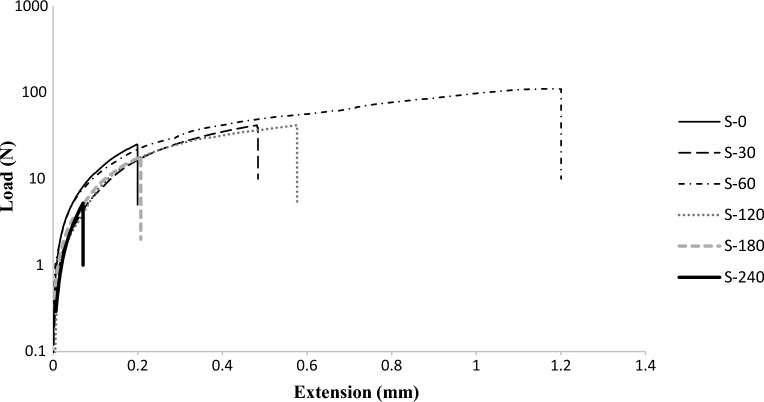
The applied force vs the elongation curve generated from the tensile test for the 6-representative samples during the bonding strength measurement.

The silanization effect on the bond strength between PDMS and LiNbO_3_ was assessed using a tensile test. The tensile test measures the bonding between the PDMS microchannel and the LiNbO_3_ wafer. Tests were conducted on a total of 30 samples, with five samples of each of the six representative samples. Each sample comprises a 15 mm by 15 mm square PDMS component bonded to a LiNbO_3_ substrate. Figure 8 depicts the arrangement of the tensile testing machine and specimens. Each sample consists of three parts: PDMS attached to LiNbO_3_ wafer, adhesive layer between sample and sample holder, and 3D-printed sample holders. The 3D-printed sample holder was developed specifically for this experiment to facilitate the test. The sample holders supplied with the instrument are incompatible with extremely small samples and low loads. The 3D-printed sample holder was designed to fit directly into the testing machine in order to eliminate any potential for error. The test results and analysis were in close agreement with those of previous tests. Table 2 displays the results of the tensile strength test. The test reveals that the S-60 sample had a tensile strength of approximately 500 kPa. Sample S-60 exhibited the strongest adhesion out of the six representative samples. After 30 min of silanization, the bonding strength increases, as shown in the table. However, after one hour of silanization, it reaches a maximum value and then begins to decrease to values lower than that of the untreated PDMS sample. Silanization for periods exceeding two hours has a detrimental effect on the bonding strength. In general, the better the bonding strength, the better the microchannel's bonding and sealing. The load versus extension curve produced by the tensile test is depicted in Fig. 9. The slope of the lines indicates the reliability of the test, as the slopes for the six representative samples fall within the same range, indicating similar properties but distinct failure points.

**Table 2:** The results of the tensile strength test for the 6-representative samples showing the average maximum load and the average tensile strength of each sample.

Sample label	Average maximum load (N)	Average tensile strength (kPa)
S-0	25	111
S-30	42	185
S-60	110	491
S-120	42	186
S-180	18	80
S-240	5	23

Sample’s bonded surface area = 15 mm × 15 mm.

## Experiments

The silanization protocol described in this work is used to build acoustofluidic systems and then test their hermetical sealing and applications for microparticle manipulation at very high flow rates. The results of both tests are detailed in the following sections.

### Hermeticity test

**Figure 10:**
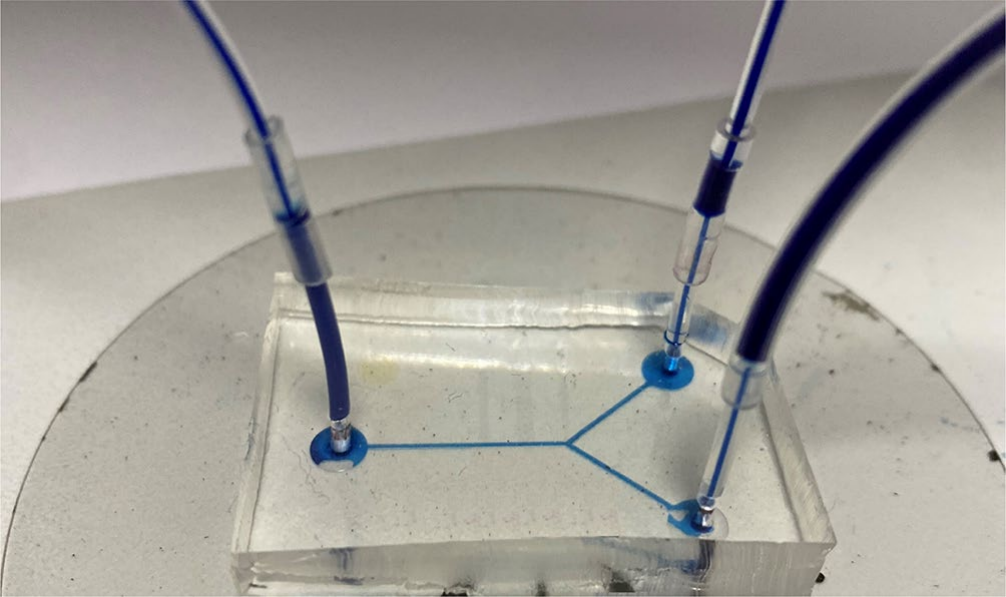
A photo of the microchannel leakage testing conducted using colored deionized water and syringe pump. The tested channel consists of one inlet at the left side and two outlets at the right side.

In the hermeticity test, a simple microchannel with one inlet and two outlets is subjected to varying water flow rates, as depicted in Fig. 10. The microchannel had dimensions of 160 µm in width, 70 µm in height, and 3000 µm in length. The microfluidic device was fabricated using the described silanization technique. The PDMS element was silanized for 60  min, as the S-60 sample demonstrated the highest bonding strength. A syringe pump was used to flow a colored solution of deionized water into the microchannel of the device, thereby testing its hermeticity. The flowrate was increased gradually from 0 to 1 L/h. which is extremely high flowrate for microfluidics. The effect of sudden pressure variation on the hermeticity of the microfluidic device was tested by implementing sudden changes in flowrate. The microchannel was able to withstand both the high flowrate and the high flowrate variations. There were no leakage issues or indications of PDMS debonding from the LiNbO_3_ wafer. It was also confirmed through repeated testing of the same device that the microdevices can be reused multiple times without degrading in performance.

Compared to other PDMS surface enhancement methods, the proposed method illustrated excellent permanent fluid sealing capabilities and good bonding results. A comparison of different surface enhancement methods is shown in Table 3. In terms of tensile strength capability, the silanization method demonstrated an average value among other enhancement methods using plasma treatment. However, in terms of sealing capabilities, the silanization method demonstrated outstanding performance, handling a flow of up to 1 L/h. This sealing capability can be explored further to be applied in different engineering applications. It is also clearly mentioned that plasma treatment methodologies are of temporary nature, whereas the silanization method demonstrated a permanent nature in terms of PDMS surface enhancement. It’s worth mentioning that this PDMS surface enhancement method can be further investigated to be applied in different fields where a high flowrate is needed.

**Table 3:** Comparison of different surface treatment methodologies to enhance the bonding between PDMS and LiNbO_3_.

Reference	Surface treatment method	Maximum tensile strength (kPa)	Maximum flowrate (μl/min)	Surface treatment status
**Current work**	**TMSPMA**	**500**	**16,700**	**Permanent**
^23^	Air plasma	350	10–20	Temporary
^23^	O_2_ plasma	1100	10–20	Temporary
^23^	N_2_ plasma	1000	10–20	Temporary
^23^	O_2_–N_2_ plasma	1400	100	Temporary

Significant values are in bold.

### Case study: particle manipulation via TSAW

This case study presents an experiment that demonstrates the use of TSAW to manipulate PS particles on an acoustic manipulation platform that was built in accordance with the prescribed treatment method. The flowrates and micro entity types of TSAW devices available in the literature are presented in Table 4. Clearly, the flowrate observed with the new surface treatment protocol is significantly higher than those reported in the literature. This means that the new method for PDMS surface enhancement is compatible with the majority of current applications using TSAW. Whereas the plasma treatment methodology covers limited applications using TSAW where the flow rate required is below 100 µL/min. The reported bonding method for PDMS and LiNbO_3_ paves the way for increased throughput in acoustofluidics applications with high flow rates. In addition, this new method will facilitate the creation of new microsystems and devices that can be utilized to facilitate and accelerate manipulation processes.

**Table 4:** Literature review of the available manipulation devices (PDMS + LiNbO_3_) that utilizes TSAW showing the different flowrates (throughput).

#	Reference/date	Particles manipulated	Throughput (μl/min)
1	^42^ 2015	PS	5
2	^18^ 2016	PS	7
3	^20^ 2017	MCF-7/blood	5
4	^43^ 2017	PS/PMMA/fused silica	2
5	^44^ 2017	PS	17
6	^45^ 2018	U-87/RBC	0.3
7	^21^ 2019	Mixing deionized water	50–400
8	^19^ 2020	PS	1
9	^46^ 2021	MCF-7/PS	11
10	^47^ 2022	PS	20

Acoustophoresis, the process of controlling particle movement by utilizing sound waves (pressure) to induce particle migration, encompasses three main types of acoustic force application in microfluidics. These methods include bulk acoustic waves, standing SAW, and TSAW. This case examines the latter phenomenon. TSAW manipulation is determined by distinct parameters, including the particle size, particle density, and compressibility of the target particles in relation to the suspending liquid. Before examining the proof-of-concept experiment, a section is dedicated to describing the related fundamentals of the TSAW particle manipulation platform^8^.

Acoustophoresis is a label-free and non-contact particle manipulation technique that enables high-throughput microdevices. Moreover, the use of TSAW has been proven as a secure method to manipulate biological samples because of its biocompatibility, which has highly impacted the development of TSAW-based acoustophoretic platforms for various types of microparticle migration in a very positive way^30, 37–39^. The term "TSAW" refers to travelling or progressive surface waves which dissipate after travelling a certain distance because of the attenuation effect. King^40^ studied the effect of acoustic radiation pressure on a rigid sphere. His research resulted in the development of an equation that permits the calculation of the radiation pressure on rigid spheres with a small circumference relative to the applied wavelength, as follows:

1 $$\overline{P }=2\pi {\rho }_{0}{\left|A\right|}^{2}{\alpha }^{6}\frac{\left\{1+\frac{2}{9}{(1-\frac{{\rho }_{0}}{{\rho }_{1}})}^{2}\right\}}{{(2+\frac{{\rho }_{0}}{{\rho }_{1}})}^{2}}+terms\, in\, {\alpha }^{8}\, and\, higher\, powers$$

where ${\rho }_{0}$ and ${\rho }_{1}$ are the medium and the sphere densities respectively. $\left|A\right|$ is the coefficient of the incident radiation field and $\alpha$ is a dimensionless parameter defined as $\alpha =ka$ , where *a* is the radius of the sphere and $k$ is the incident wave number ($k=2\pi / \lambda )$. Generally, TSAW can be simply produced by printing a single IDT on a piezoelectric wafer, in this case a LiNbO_3_ wafer, and connecting and supplying the IDT to the required power supply to create the required acoustic force. Further investigation into the acoustic radiation pressure by Hasegawa and Yosioka^41^ concluded that the sphere’s elasticity has an effect on the calculation of the acoustic radiation force. Their studies were focused only on plane progressive TSAW. They derived the following representation of the acoustic radiation force generated by TSAW on an elastic sphere as:

2 $${F}_{TSAW}={Y}_{T}\pi {a}^{2}E$$

where ${F}_{TSAW}$ is the time averaged acoustic radiation force by TSAW,${Y}_{T}$ is a dimensionless parameter called the acoustic radiation force factor defined as the acoustic radiation force per unit acoustic energy density per unit cross sectional area of the microsphere, $a$ is the radius of the sphere, and $E$ is the mean energy density.

**Figure 11:**
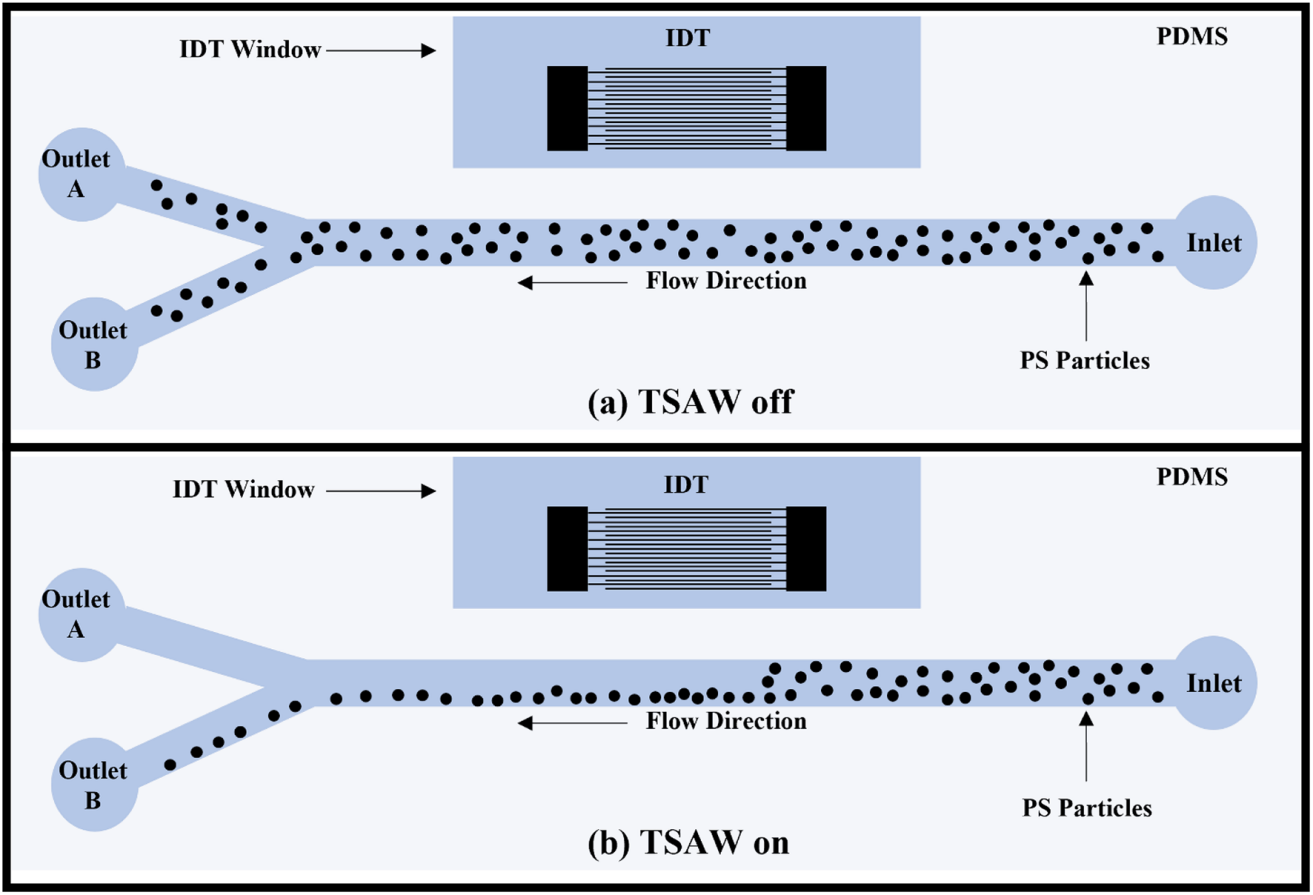
Schematic illustration of the TSAW based particle manipulation platform. (**a**) TSAW off: particles are flowing everywhere along the channels width and leaving the channel from both outlets. (**b**) TSAW on: particles are pushed to the channel’s wall away from the IDT and particles are leaving the channel trough outlet B only.

**Figure 12:**
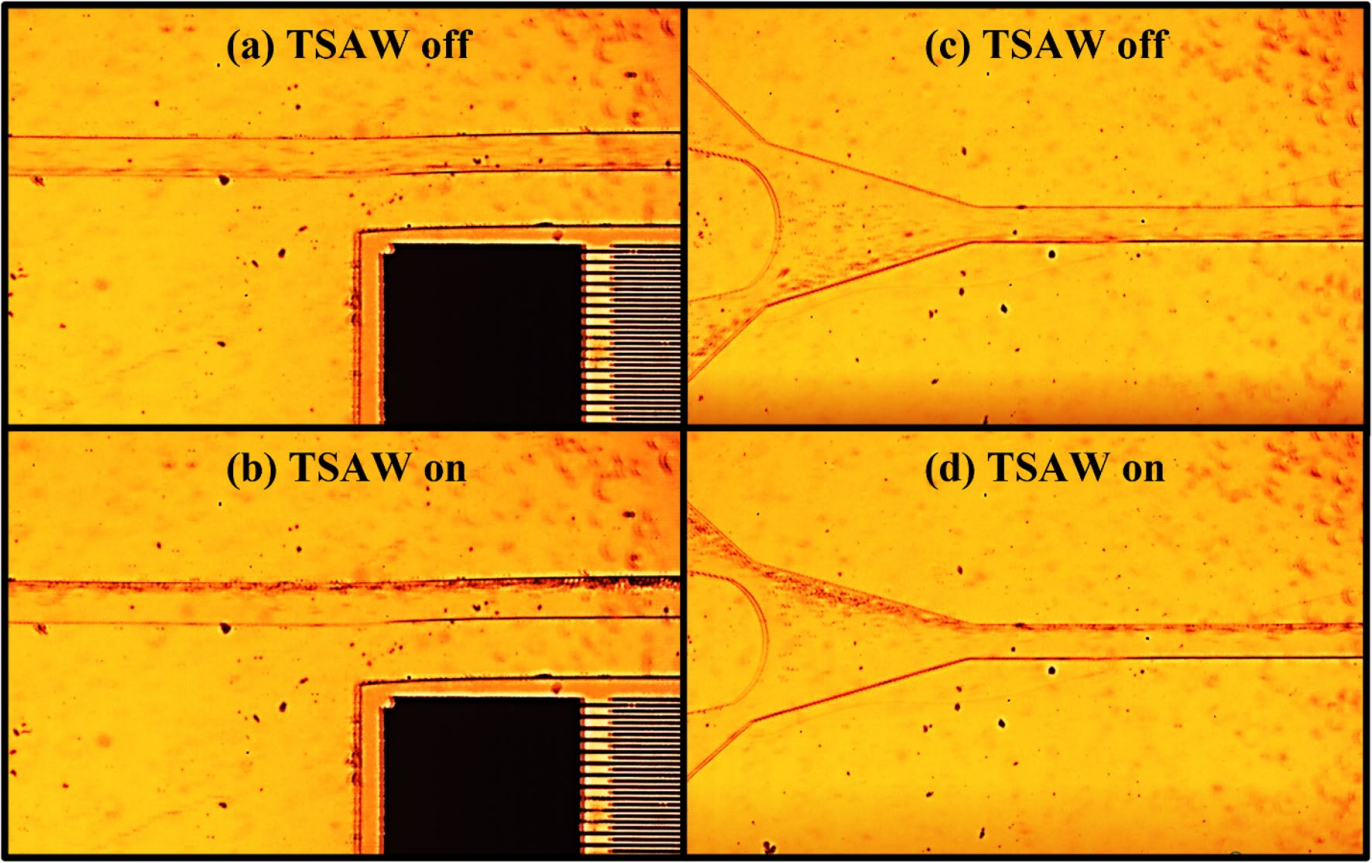
PS particles manipulation experiment as observed under an inverted microscope. (**a**) The location is at the middle of the microfluidic device. The TSAW device is off. The PS particles are flowing everywhere along the width of the channel at flow rate of 1000 µL/h. (**b**) The location is at the middle of the microfluidic device. The TSAW device is on. The PS particles are pushed toward the opposite channel’s wall due to the acoustic radiation force. (**c**) The location is at the outlet of the microfluidic device. The TSAW device is off. The PS particles are exiting the channel through both outlets at flow rate of 1000 µL/h. (**d**) The location is at the outlet of the microfluidic device. The TSAW device is on. The PS particles are exiting from the upper outlet only because of the effect of the acoustic radiation force.

Similar to the hermeticity test, the PDMS was silanized for sixty minutes as it showed the best results in terms of bonding strength and hermeticity. Figure 11 illustrates schematically the manipulation platform and the working principles. In Fig. 11a, the particles are moving while the IDT is not receiving a signal to generate the necessary acoustic force. In this instance, particles are exiting both outlets. Figure 11b depicts the anticipated result of manipulating PS particles with TSAW. As TSAW pushes the particles away, it is anticipated that they will flow near the far wall. Particles will only exit the channel through outlet B. As depicted in Fig. 12, the TSAW particle manipulation experiment was conducted and visualized under an inverted microscope (Zeiss Axio Observer) equipped with a high-speed camera (Fastcam SA-X2 from Photron) to monitor the manipulation process. PS particles (from micro–Particles GmbH) with a diameter of 6.69 µm were suspended in deionized (DI) water (suspension medium) and then pumped into the microchannel using a syringe pump at a flow rate of 1 mL/h. This high flow rate was selected to demonstrate the microchannel's ability to withstand high flow rates without leaking. To prevent PS particles from adhering to the channel's walls, a drop of Tween-20 was added to the solution. Once the flow became stable within the microchannel, a waveform generator was used to apply an AC signal with a frequency of 100 MHz (IDT resonant frequency) and a voltage amplitude of 5 V peak-to-peak to the IDT array (RIGOL DG4102). Signal amplification was performed using a power amplifier (ZHL-1-2W-N+). The device was able to manipulate PS particles and direct them toward the opposite channel's wall. The same device has been tested multiple times at various flow rates with no leakage observed.

## Conclusion

In conclusion, a novel surface treatment technique has been developed to permanently enhance the bonding and hermeticity of acoustofluidic devices. Using the TMSPMA silane surface modification reagent, a detailed implementation protocol for the surface treatment has been described. The method does not require extremely high temperatures or pressure to be applied. Compared to other temporary plasma-based treatments, the demonstrated method enabled a permanent enhancement of PDMS hydrophilicity. Multiple characterization tests were conducted to examine the effect of the treatment method on the PDMS surface. The results of the tests indicated that a treatment duration of one hour using TMSPMA is optimal. The hydrophilicity of PDMS has been shown to be permanently and significantly improved after one hour of silanization. In addition, the treatment positively affected the morphology of the PDMS surface, while the bonding strength was increased by more than twofold. The maximum sealing pressure of approximately 500 kPa was reached for the sample that was treated for one hour. The PDMS surface energy exhibited a noticeable increase. These improvements demonstrate that the newly introduced method is very promising and can aid in creating acoustofluidic devices. Experiments demonstrating proof-of-concept have illustrated that the fabricated devices can withstand a flow rate of up to 1 L/hr without leaking or debonding, which covers TSAW-based and other microfluidic applications. With particles being manipulated at a flow rate of 1 mL/hr via TSAW, the demonstration experiment of the TSAW-based particle manipulation device showed promising improvements in boning and sealing. It has been demonstrated that the introduced PDMS surface treatment method permanently improves the bonding efficiency and bonding strength of PDMS and LiNbO_3_. Microfluidic devices based on SAW technology can make extensive use of this improved performance.

## Data Availability

All data generated or analyzed during this study are included in this published article.
